# Hydroxyapatite Nanopowders for Effective Removal of Strontium Ions from Aqueous Solutions

**DOI:** 10.3390/ma16010229

**Published:** 2022-12-27

**Authors:** Silviu Adrian Predoi, Steluta Carmen Ciobanu, Mariana Carmen Chifiriuc, Mikael Motelica-Heino, Daniela Predoi, Simona Liliana Iconaru

**Affiliations:** 1 Département de Physique, École Normale Supérieure Paris-Saclay, 4 Avenue des Sciences, 91190 Gif-sur-Yvette, France; 2 Physique Fondamentale, Université Paris-Saclay, 3 Rue Joliot Curie, 91190 Gif-sur-Yvette, France; 3 Life, Environmental and Earth Sciences Division, Research Institute of the University of Bucharest (ICUB), University of Bucharest, 060023 Bucharest, Romania; 4 National Institute of Materials Physics, Atomistilor Street, 077125 Magurele, Romania; 5 Department of Microbiology, Faculty of Biology, University of Bucharest, 1-3 Aleea Portocalelor Str., District 5, 060101 Bucharest, Romania; 6 Biological Sciences Division, The Romanian Academy, 25, Calea Victoriei, 010071 Bucharest, Romania; 7 Department of Civil Engineering and Environment, Université d’Orléans, ISTO, UMR 7327 CNRS, 1A Rue de la Férollerie, 45071 Orléans, France

**Keywords:** hydroxyapatite, powder, strontium, decontamination, biological properties

## Abstract

Drinking water contamination has become a worldwide problem due to the highly negative effects that pollutants can have on human organisms and the environment. Hydroxyapatite (HAp) has the appropriate properties for the immobilization of various pollutants, being considered amongst the most cost-effective materials for water decontamination. The main objective of this study was to use synthesized hydroxyapatite for the elimination of Sr^2+^ ions from contaminated solutions. The hydroxyapatite used in the decontamination process was synthesized in the laboratory using an adapted method. The hydroxyapatite powder (HAp) resulting from the synthesis was analyzed both before and after the elimination of Sr^2+^ ions from contaminated solutions. The efficiency of the HAp nanoparticles in removing Sr^2+^ ions from contaminated solution was determined by batch adsorption experiments. X-ray diffraction (XRD), energy dispersive X-ray spectroscopy (EDX), scanning electron microscopy (SEM) and Fourier transform infrared spectroscopy (FTIR) were used to study the HAp samples before and after the removal of Sr^2+^ ions. The ability of HAp nanoparticles to eliminate strontium ions from contaminated solutions was established. Moreover, the removal of Sr^2+^ ions from the contaminated aqueous solutions was highlighted by ultrasound measurements. The value of the stability parameter calculated by ultrasonic measurements after the removal of Sr^2+^ ions from the contaminated solution was similar to that of double distilled water whose stability was used as reference. The outcomes of the batch experiments and the parameters obtained from Langmuir and Freundlich models indicated that the HAp nanoparticles had a strong affinity for the elimination of Sr^2+^ ions from polluted solutions. These results emphasized that HAp nanoparticles could be excellent candidates in the development of new technologies for water remediation. More than that, the outcomes of the cytotoxic assays proved that HAp nanoparticles did not induce any noticeable harmful effects against HeLa cells and did not affect their proliferation after 1 day and 7 days of incubation.

## 1. Introduction

Today, one of the most important problems that affects the public health sector around the world is environmental pollution (water, soil, air, etc.,) with heavy metals. It is well known that such metals are not biodegradable and tend to accumulate in living organisms [1]. Moreover, the presence of higher quantities of metals in the water/soil leads to severe diseases and can irreversibly endanger environmental ecosystems [2,3,4]. In this context, the elaboration of new materials with enhanced properties for environmental remediation are of great interest. Previous studies have proved that hydroxyapatite (Ca_10_(PO_4_)_6_(OH)_2_, HAp), which is the major inorganic constituent found in human bones and teeth, has the ability to interact with various ions by an adsorption/ion exchange process [5]. Therefore, due to these excellent properties, in recent years, HAp nanoparticles have been extensively studied for the adsorption of different pollutants from contaminated environments [6,7].

Countries such as India, the U.S.A, and Japan have reported strontium contamination of various water sources (including groundwater) [5] which represents an important public health problem, as the ingestion of a high concentration of strontium is known to have harmful effects for living organisms [5]. Additionally, strontium is used for the manufacturing processes of cathode ray tubes and in the pigment industry [8]. Therefore, the main source of water contamination with strontium could be represented either by industry and/or nuclear wastewaters [8,9]. On the other hand, the main source of strontium for humans is represented by vegetables and cereals (the daily recommended strontium dose recommended is 2–4 mg) [10]. Furthermore, due to their similarity to Ca^2+^ ions from the HAp structure, Sr^2+^ ions could be easily adsorbed in bone tissue after ingestion [9,11]. Long-term consumption of water contaminated with strontium can favor the appearance of diseases such as osteosarcoma and/or other neurological disorders [5,9]. The conclusions of the studies carried out on embryo–larval zebrafish by Z. Liu and coworkers [12] revealed that a high concentration of Sr^2+^ (0.21 mg/mL) presented hepatotoxic effects with the main manifestations of hepatomegaly [12]. Ozgur et al. [13] illustrated in their work in Turkey that the presence in the soil of a strontium concentration higher than 350 ppm may represent one of the causes of rickets [13]. Thus, it is mandatory to keep the strontium concentration in water/soils at the lowest possible concentrations (under 4.0 mg L^−1^ of strontium in drinking water) [5].

In the last years, water decontamination treatment has been carried out using physicochemical methods (e.g., adsorption, sedimentation, chemical precipitation, nanofiltration, electrochemical treatment and reverse osmosis), pump and treat test or biological methods [14,15,16,17]. Using coagulation/filtration techniques, a low adsorption efficiency of strontium from drinking water (around 15% according to [16,18]) was obtained. According to Najm I. [19], the most feasible methods for strontium radionuclide removal from drinking water are represented by the use of chemical precipitation together with ion-exchange techniques. Another efficient technology used in water decontamination treatments is represented by membrane technologies and is expected to exceed 95% efficiency of strontium removal from wastewater [16,19]. One of the main disadvantages of these methods is represented by the high costs (for maintenance and operation). Therefore, the development of new inorganic/organic adsorbents that are cost-effective and highly efficient in the decontamination of polluted waters represents an attractive research topic at a global level [17]. Recent studies illustrated that hydroxyapatite has the ability to remove various ions (cadmium, fluoride, lead, copper, etc.,) from contaminated solutions with high efficiency [20,21,22,23,24]. This ability may be due, on the one hand, to the specific area of HAp (a growth in adsorption efficiency was remarked once in conjunction with the increase in the HAp specific surface area) [25]. On the other hand, given the HAp affinity, various types of cations could be substituted for the calcium ions (from the HAp structure) [15]. In the study conducted by Vila M. and colab. [23], it was highlighted that the adsorption capacity of Pb^2+^, Cu^2+^ and Cd^2+^ ions from polluted water is higher in the case of hydroxyapatite-biopolymer foams [23]. Recent studies made of bacterially produced hydroxyapatite (BHAP) showed that this material possesses superior adsorption properties of Sr^2+^ and Co^2+^ from aqueous solutions and groundwater than synthetic hydroxyapatite [26].

Thus, HAp could be considered a good candidate for applications in environmental remediation due to its abundance and unique properties (e.g., acid-based ion exchange capacity, thermal stability, etc.) [15,25]. Furthermore, another advantage of using HAp in environmental remediation applications is that it has the ability to be reused without reducing its functionality and is a cost-effective and ecofriendly biomaterial [15,25].

More than that, it is well known that the biocompatibility of hydroxyapatite has been extensively explored [27]. In this context, one of the cell lines used in the cytotoxicity studies is represented by cervical adenocarcinoma epithelial cells (HeLa) [28,29,30]. The results of the research reported by K. Kadu et al. [31] highlighted that the concentration and HAp nanoparticle morphology influence their cytotoxic effect on HeLa cells.

The main objective of this study was to obtain HAp nanopowders by an adapted co-precipitation method developed in the laboratory. The novelty of this study consists in the valuable information obtained using ultrasonic measurements about the stability of water contaminated with Sr ions and water after the removal of Sr ions being reported for the first time. The adsorption efficacy of HAp powders of strontium ions from contaminated solutions was estimated using non-destructive ultrasound studies. The morphology and chemical composition of the as-obtained HAp samples were studied before and after the removal of strontium ions. Furthermore, information regarding the adsorption efficacy of HAp of strontium ions from contaminated aqueous solutions was also obtained by conducting batch adsorption experiments. In addition, biological assays regarding the cytotoxicity of HAp nanoparticles, strontium-contaminated and decontaminated aqueous solutions were performed and reported for the first time.

## 2. Materials and Methods

### 2.1. Materials

Calcium nitrate tetrahydrate (Ca(NO_3_)_2_·4H_2_O), diammonium phosphate (NH_4_)_2_HPO_4_, sodium hydroxide (NaOH) were acquired from Alpha Aesare and used without further purification [32].

### 2.2. Synthesis

Hydroxyapatite was synthesized by a simple co-precipitation developed in the laboratory. The necessary quantities of Ca(NO_3_)_2_·4H_2_O and (NH_4_)_2_HPO_4_ were used in order to obtain 100 mL of each solution. Both solutions were prepared in 0.7 M NaOH at room temperature by stirring on a magnetic stirrer (Velp Scientifica, Usamti, Italy) for 3 h. The solution containing Ca(NO_3_)_2_·4H_2_O was added dropwise slowly (about 3 mL/min) to the (NH_4_)_2_HPO_4_ solution. To achieve a stoichiometric ratio, Ca/P was kept at 1.67. During the synthesis, the pH was kept constant at 14. The final mixture was continuously stirred for 6 h at a set temperature of 30 °C on a magnetic stirrer. Finally, the obtained suspension was centrifuged at 5000 rpm for 15 min. The white precipitate was washed 5 times. After each wash the obtained suspension was centrifugated and redispersed in deionized water. The precipitate obtained after the last centrifugation was dried at a temperature of 100 °C for 24 h in an oven, in air. The hydroxyapatite powder resulting from drying was used in the decontamination process.

### 2.3. Characterization Methods

The crystal structure of HAp powders and their phases were investigated by X-ray diffraction (Rigaku SmartLab 3 kW (Rigaku, Tokyo, Japan) apparatus, using CuKα radiation (λ = 1.5418 Å) and an incidence angle of 0.5°). The XRD diffractograms were obtained in the 2 theta range 15–65°. The step size was 0.001 (2 theta).

The microstructure of hydroxyapatite powders (before and after decontamination experiments) was investigated using HITACHI S4500 (Tokyo, Japan) equipment. For the chemical composition, the microscope was equipped with an X-ray energy dispersive spectroscopy (EDX) (Hitachi, Tokyo, Japan) device.

FTIR studies were conducted with the aid of a Perkin Elmer SP-100 spectrometer (Waltham, MA, USA). For this study, the spectrometer was operated in the ATR (attenuated total reflectance) mode. The ATR-FTIR spectra were recorded in the 450–4000 cm^−1^ spectral range and the samples were studied without further preparation.

The adsorption of strontium ions onto HAp nanoparticles from aqueous solutions was analyzed with the aid of batch adsorption experiments by employing a previously described procedure [1]. The studies were carried out using silicon tubes and strontium-contaminated aqueous solutions having a Sr^2+^ concentration ranging from 0 to 100 mg L^−1^. For the batch adsorption experiments, a 0.2 g amount of HAp nanoparticles were added to several contaminated solutions with various Sr^2+^ concentrations. The contaminated solutions and HAp nanoparticles were kept in contact and stirred for 24 h. During the experiment, the volume of the mixture was kept at 20 mL and the pH value was adjusted to 6. After 24 h, the tubes were centrifuged at 10,000 rpm for 45 min and the filtered supernatant was recovered and examined using flame atomic absorption spectrometry (AAS) using a Zeeman HITACHI Z-8100 instrument at a wavelength of 460.7 nm according to the working conditions for strontium. The adsorption percentage, R(%), was determined using the equation $R\left(\%\right)=\frac{C_{o}-C_{e}}{C_{0}}\times 100$, where *C_o_* and *C_e_* are the initial and equilibrium concentrations of Sr^2+^ (mg/L) ions. The kinetics involved in the adsorption of the Sr^2+^ onto HAp nanoparticles were evaluated using the adsorption models described by Langmuir and Freundlich [33,34]. The quantity of adsorbed Sr^2+^ ions onto the adsorbent at equilibrium, *Q_e_* (mg/g), was determined using the Langmuir equation $Q_{e}=\frac{\left(C_{o\;}-C_{e}\right)}{m}\cdot V$, where *C_o_* and *C_e_* expressed in mg/L depict the initial and equilibrium Sr^2+^ concentrations in mg/L, *V* (L) represents the volume of the solution and m (g) stands for the mass of the HAp nanoparticles used in the adsorption experiment. Information regarding the adsorption process was also obtained by determining the Langmuir constants, *q_m_* and *K_L_* representing the maximum adsorption capacity, and the constant energy associated with the heat of adsorption, from the graphical representation of the linear Langmuir equation, $\frac{C_{e}}{Q_{e}}=\frac{1}{\left(q_{m}\cdot K_{L}\right)}+\frac{C_{e}}{q_{m}}$ [33]. Additional information about the adsorption process was determined from the Freundlich isotherm experimental model, Qe=kf·Ce1n, by obtaining the Freundlich parameters *k_f_* and n, representing the adsorption capacity and the adsorption intensity of the adsorbent, from the graphical representation of the linear form of the Freundlich equation $lnQ_{e}=lnk_{f}+\frac{1}{n}lnC_{e}$ [34].

The stability of the water contaminated with Sr ions and after the removal of the Sr ions was evaluated by ultrasonic studies in accordance with previous studies [35,36,37].

### 2.4. In Vitro Cytotoxicity Assay

The cytotoxicity evaluation of the HAp nanoparticles was achieved in vitro using the MTT (3-4,5-Dimethylthiazol 2,5-diphenyltetrazolium bromide) test. The cytotoxicity assay was carried out using HeLa cell line as previously reported [38,39]. The viability of the HeLa cells was determined after being treated for 1 day and 7 days with HAp nanoparticles and also with strontium-contaminated and decontaminated aqueous solutions. The cell viability was determined from the absorbance of the suspensions at a wavelength of 595 nm by a TECAN spectrophotometer (Tecan GENios, Grödic, Germany) instrument. The MTT assays were carried out in triplicate and the results are shown as mean ± SD (standard deviation). Furthermore, the morphology of the HeLa cells put into contact for 1 day and 7 days with the contaminated and solutions decontaminated by employing HAp nanoparticles was also studied by optical microscopy.

## 3. Results

**Figure 1:**
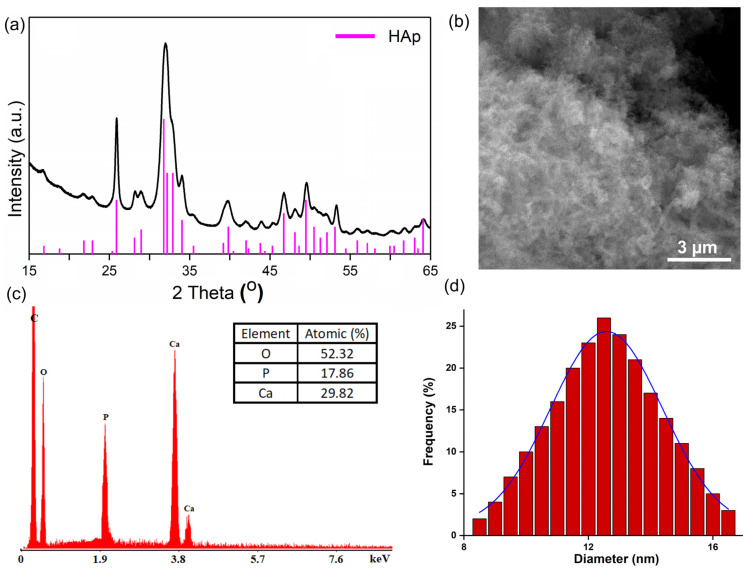
XRD patterns of synthesized powder (**a**); SEM image of HAp powder (**b**); EDX spectra of HAp (**c**) and SEM particle size distribution (**d**).

The X-ray diffraction technique was used to determine the phases of powders used in the batch adsorption experiments before and after the removal of Sr ions. Figure 1a reveals the XRD patterns of the synthesized powder used in the removal of Sr ions from contaminated aqueous solutions. The major peaks identified at 2θ showed that the synthesized powder was assigned to the pure apatite phase (JCPDS No.9-432). No visible peaks of a secondary phase formation were found. The average particle size of HAp nanoparticles was calculated with the Debye–Scherrer formula [40]. For the XRD mean particle size, a value of 11 nm was obtained (for the HAp powders). The SEM images obtained for the HAp powder revealed particles at a nanometric scale with ellipsoidal morphology (Figure 1b). The mean particle size obtained from the SEM particle size distribution was 12.5 nm (Figure 1c). In the EDX spectra, specific to hydroxyapatite powders (Figure 1d), only the presence of calcium (Ca), phosphorus (P) and oxygen (O) could be observed.

**Figure 2:**
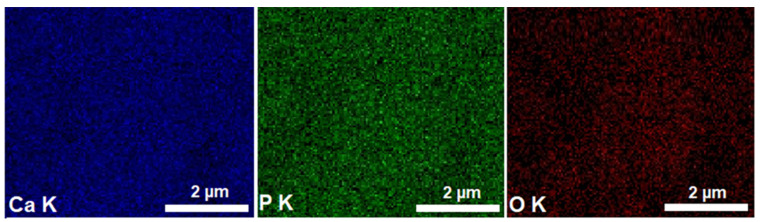
Elemental distribution maps obtained of HAp samples.

The elemental distribution maps obtained of the HAp samples are presented in Figure 2. In Figure 2, the uniform and homogenous distribution of the main chemical constituents (Ca, P and O) in the HAp nanopowder can be noted.

The results of the XRD, SEM, EDX and elemental mapping studies following the evaluation of the HAp powders recovered after the process of removing strontium ions from the contaminated solutions are also presented. The role of these studies carried out on the HAp powders recovered after the adsorption of strontium ions from the contaminated solutions was to highlight their efficiency in the decontamination process.

**Figure 3:**
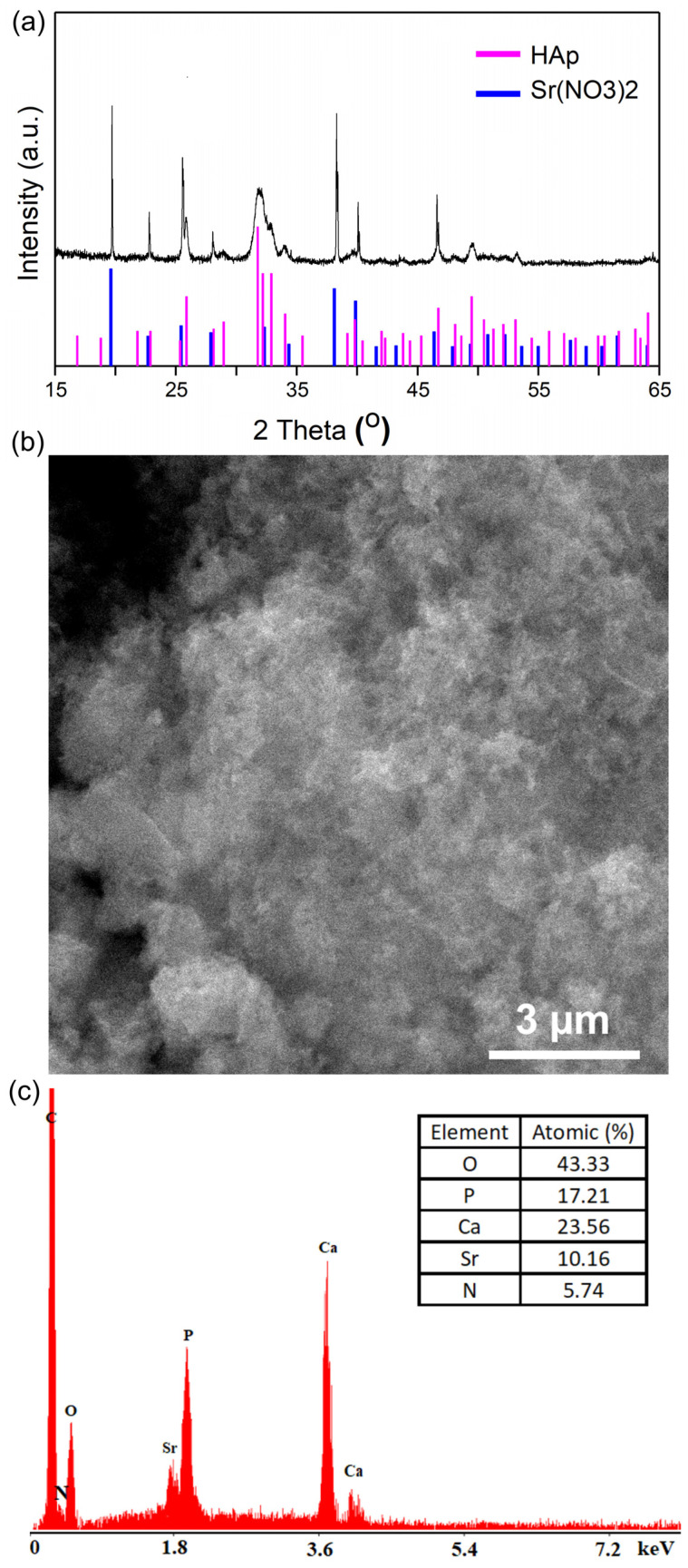
XRD patterns (**a**), SEM micrographs (**b**) and EDX spectra (**c**) of synthesized powder after removal of strontium ions from contaminated solution.

Figure 3a presents the XRD patterns of the powder recovered after the removal of Sr ions from the contaminated solutions. It can be seen that the recovered powders are composed of a main phase attributed to pure hydroxyapatite and a secondary phase (Figure 3a). A small broadening and a slight shift of the peaks characteristic of pure HAp could be due to the incorporation of the Sr^2+^ ions into the crystal structure of HAp, which is in full concordance with the results reported by Bogya et al. [41]. On the other hand, in Figure 3a, major peaks (such as 19.63, 22.73, 25.44, 27.92, 38.19, 39.87, 46.43 and 52.28°) that indicate the presence of strontium can be seen. The secondary phase was attributed to strontium nitrate (Sr(NO_3_)_2_, JCPDS No. 71-3823). The maxima belonging to strontium nitrate could be due to doping on the HAp surface. The broadening of the maxima observed in the diffractogram could be due to the substitution or the incorporation of the Sr^2+^ ions into the crystal structure of HAp. Another explanation for the broadening of the peaks could be given by the nanometric dimension of the particles. Similar results were reported in recent studies presented by Nie et al. [42]. The presence of strontium in the recovered powders after the removal of strontium ions from the contaminated solutions revealed that the synthesized powder of HAp was effective in the decontamination process.

The results of the SEM studies on the hydroxyapatite powders after the removal of strontium ions are presented in Figure 3b. It should be noted that the particles preserve their nanometric size. Moreover, our results suggest that the ellipsoidal morphology of the nanoparticles suffered a slight change after the decontamination experiments (Figure 1b and Figure 3b).

The EDS measurement results depicted in Figure 1d and Figure 3c suggest that the samples are pure. In the Figure 3c, besides the specific elements of the hydroxyapatite structure, the presence of a strontium line could be observed, a fact that suggests the adsorption of strontium ions by the hydroxyapatite powders. The presence of the carbon line is due to the use of double-sided carbon tape in the sample preparation for SEM observation.

**Figure 4:**
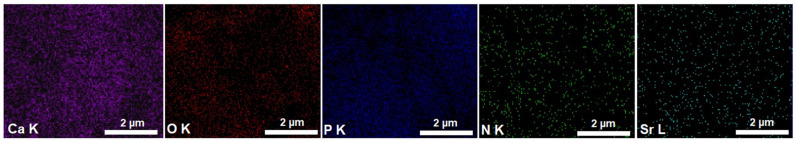
Elemental distribution maps obtained of HAp samples after the decontamination experiments.

The elemental distribution maps obtained of the HAp samples after the strontium adsorption experiments are presented in Figure 4. In Figure 4, the presence and uniform distribution of strontium (Sr) is highlighted in the samples. Furthermore, in the elemental distribution map, the homogeneous distribution of phosphorus (P), calcium (Ca), nitrogen (N) and oxygen (O) in the studied powders can be noted.

**Figure 5:**
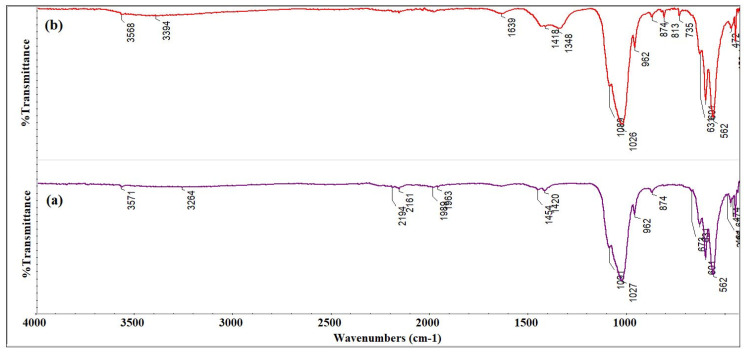
The Fourier transform infrared spectra characteristics of the HAp samples before (**a**) and after (**b**) the decontamination experiments.

Furthermore, FTIR studies were carried out on HAp powders before and after the elimination of Sr^2+^ ions from the contaminated aqueous solutions. Figure 5a,b illustrates the FTIR spectra obtained of the HAp samples (before and after the elimination of strontium ions from the contaminated aqueous solution). The band noticed at 631 cm^−1^ was attributed to the δ OH^−^ vibration, and it is a characteristic of the HAp structure [37,43,44]. According to the studies reported by Y. Y. Ozbek et al., the vibrational band observed at 874 cm^−1^ can be attributed to the presence of HPO_4_^−^ [37,43,45] in the samples. On the other hand, the presence of adsorbed water in the HAp powders is underlined by the vibrational bands noted at about 1639 cm^−1^ and at 3571 cm^−1^ [37,43]. The main bands found at 1091, 1027, and 961 cm^−1^ belong to the vibration modes of the phosphate groups (ν_3_ and ν_1_) [37,43,44]. The two vibrational bands observed at 601 and 562 cm^−1^ are also characteristics of (ν_4_) of the phosphate groups [37,43,44].

As can be seen, in the FTIR spectra obtained for the HAp sample recovered after the batch experiments (Figure 5b), besides the presence of the main vibrational bands at 962, 601, 562, 1026 and 1089 cm^−1^ that are characteristic of the phosphate groups (ν_1_, ν_4_ and ν_3_) of the HAp structure [37,43], the presence of a band at 1348 cm^−1^ was noted, that was given by the presence of a N–O vibration (from the Sr(NO_3_)_2_ structure) [46]. Additionally, the vibrational bands observed at 735 and 813 cm^−1^ belong to the bending mode of ${\mathrm{NO}}_{3}^{-}$ from the Sr(NO_3_)_2_ structure [46]. Thus, the obtained FTIR results were in good agreement with the XRD results suggesting the presence of a secondary phase in the hydroxyapatite powder recovered after the decontamination experiments.

**Figure 6:**
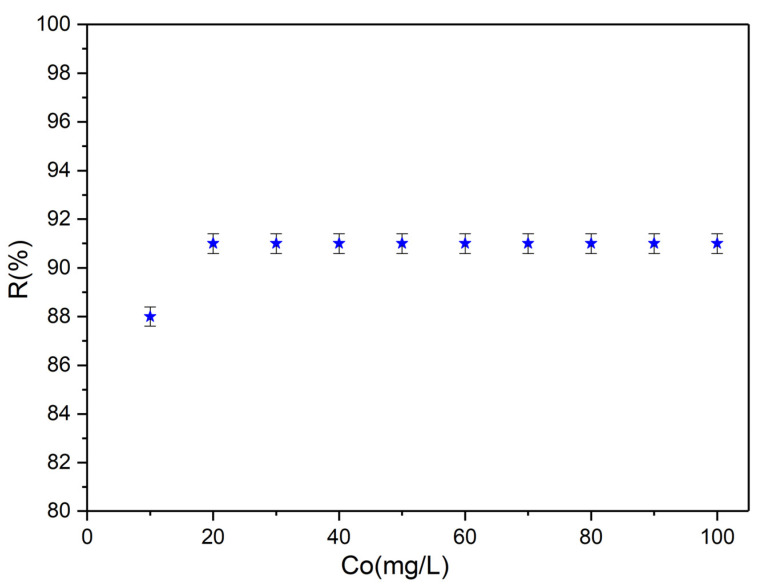
The removal percentage of Sr^2+^ ions from contaminated aqueous solutions using HAp nanoparticles.

The adsorption of strontium ions from aqueous solutions by HAp nanoparticles was investigated by flame atomic absorption spectroscopy. The batch adsorption experiments were carried out in triplicate and at ambient temperature. The removal efficiency of Sr^2+^ ions using HAp nanoparticles was determined using the results obtained from the batch adsorption experiments. Figure 6 presents the percentage of removal efficiency (R%) and its correlation with the initial concentration of Sr^2+^. The results demonstrated that the removal efficiency of strontium ions using HAp nanoparticles was higher than 90% and was strongly correlated with the initial Sr^2+^ concentration. The outcomes also underlined that the HAp nanoparticles own a strong affinity for Sr^2+^ ions, having a percentage removal efficiency (R%) of 91% for an initial Sr^2+^ concentration above 20 mg/L.

**Figure 7:**
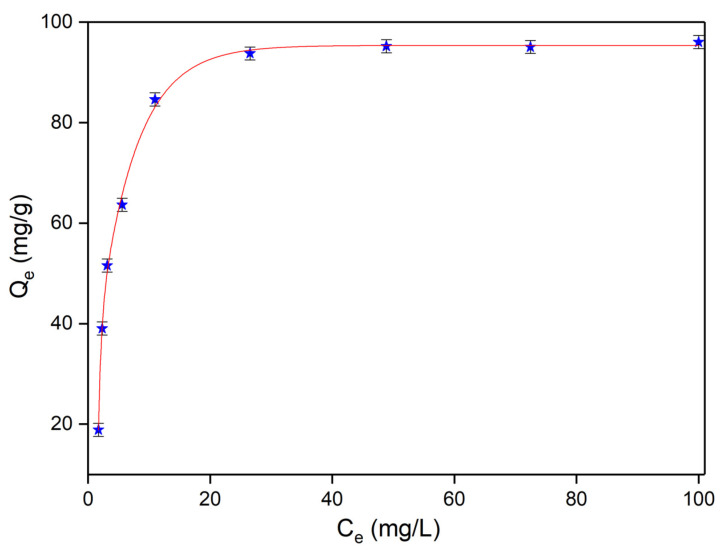
Graphical representation of the amount of Sr^2+^ ions adsorbed at equilibrium by HAp nanoparticles.

The kinetics involved in the adsorption processes of strontium ions by HAp nanoparticles were described using both Langmuir and Freundlich adsorption models [47,48]. The Langmuir adsorption model was first elaborated for describing the activated carbon gas–solid adsorption phase, and since then, has been used for the evaluation of the efficiency of many materials [49]. The model proposed by Langmuir described the theory for a monolayer adsorption and depicted a process of adsorption that could only happen for a finite number of localized and previously defined areas, which were identical and equivalent [49,50]. The experimental data fit, using the theoretical Langmuir model for the strontium ions adsorption from contaminated solutions by HAp nanoparticles, is depicted in Figure 7.

**Figure 8:**
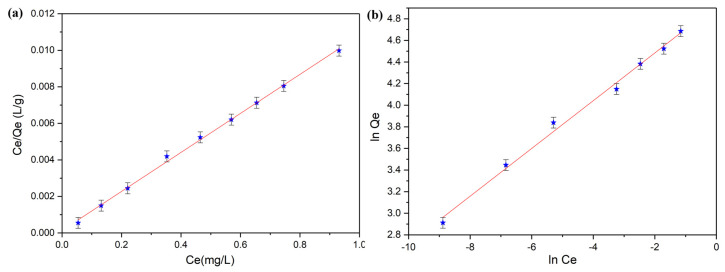
Langmuir (**a**) and Freundlich (**b**) graphical linearized equations for the adsorption of strontium ions on HAp nanoparticles.

The data derived from the batch adsorption experiments emphasized that HAp nanoparticles were highly efficient in removing Sr^2+^ ions from contaminated solutions. The results determined that both the Langmuir and Freundlich isotherm models had R^2^ values equal or higher than 0.99 which showed a good fit with each model. However, the data showed that the experimental data was better simulated by the Langmuir model (R^2^ = 0.997) compared to the Freundlich (R^2^ = 0.993). The graphical linearized equations for the adsorption of strontium ions on HAp nanoparticles are depicted in Figure 8.

The Langmuir constants calculated from the AAS data and using the Langmuir adsorption model revealed a value of 93.63 ± 3.25 mg (Sr)/g for the adsorption capacity and a value of 78.19 L/mg for the *K_L_* coefficient. Moreover, the Freundlich model was also used to better understand the mechanisms involved in the removal of strontium ions using HAp nanoparticles. The Freundlich adsorption model implies, that, if the Freundlich constant, n, is equal to 1, then the separation of the two phases is independent of the concentration, while a 1/n value below 1 depicts a normal adsorption process and a value of 1/n less than 1 indicates a cooperative adsorption process [51]. The values obtained for the Langmuir and Freundlich parameters from the adsorption batch experiments are presented in Table 1.

The results highlighted that the value for constant n, determined using the linearized Freundlich equation, for the Sr^2+^ ion adsorption onto HAp nanoparticles, was above 1. The results from the experimental data showed a value of 1/n below 1, which suggested a normal adsorption process in the case of strontium ion adsorption onto HAp nanoparticles. Additionally, the data underlined that the adsorption of Sr^2+^ onto HAp nanoparticles at room temperature and at a pH value of 6 was high, due to a relatively large affinity that exists between the Sr^2+^ and HAp nanoparticles. These results may be explained by the fact that Sr^2+^ can be adsorbed onto the HAp surface by the substitution mechanism of Ca^2+^ for Sr^2+^ [52,53]. Even though the exact mechanisms that influence the adsorption of heavy metals onto different types of nanoparticles are yet to be completely understood, possible mechanisms have been proposed over the years. More than that, it has been reported that the adsorption could be influenced by different parameters, such as the solution pH [54], the temperature at which the studies are conducted [55], the physicochemical parameters of the HAp nanoparticles [56], and also the presence of other metal ions in the solution [20]. The possible mechanisms involved in Sr^2+^ adsorption onto HAp have been proposed as a surface adsorption or substitution in the HAp crystal lattice [5]. Moreover, both Rosskopfova et al. [52] and Sekine et al. [53] reported that Sr^2+^ may be adsorbed onto the HAp surface through the substitution of Ca^2+^ for Sr^2+^ ions due to the similarity of their ionic radii; the ionic radius of Sr^2+^ is found between 0.09 and 1.3 nm, while the ionic radius of Ca^2+^ is 1.2 nm, which is responsible for the adsorption of Sr^2+^ onto the surface of the hydroxyapatite, which led to the ionic substitution of Ca^2+.^.

The outcomes of the batch experiments and the data obtained employing both Langmuir and Freundlich models indicated that the HAp nanoparticles exhibited a strong affinity for the adsorption of Sr^2+^ ions from contaminated solutions. These results emphasized that HAp nanoparticles may be excellent candidates in the development of new technologies for water remediation.

**Figure 9:**
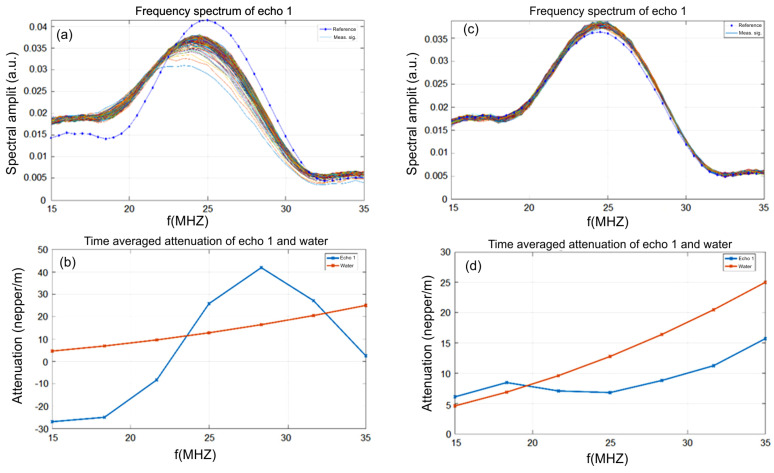
Frequency spectrum of echo 1 before (**a**) and after strontium removal (**c**). Time-averaged attenuation of echo 1 and water before (**b**) and after strontium removal (**d**).

Moreover, the removal of Sr^2+^ ions from the contaminated aqueous solution was highlighted by ultrasound measurements. Ultrasonic studies can provide important information about suspensions and solutions because the velocity of ultrasound through suspensions and solutions in the linear approximation of small disturbances depends on the average density and average compressibility. This study presents the experimental results of ultrasonic measurements in bidistilled water, Sr^2+^-contaminated water and water after decontamination, by the temporal analysis of the signal which determines the temporal deviations between the equivalent echoes in different fluids with an accuracy of 1 ns. The efficiency of the HAp powder in removing strontium from water solution is also demonstrated in Figure 9a. The time-averaged evolution of the spectral attenuation of echo 1 is presented in Figure 9a. The attenuation peak at 25 MHz in water was at 27 Np/m (Figure 9b) and after removing the strontium, the maximum attenuation at 25 MHz was noticed at 7 Np/m, a value that it is very close to that of bidistilled water (Figure 9d). Moreover, in the case of the polluted water, a minimum attenuation at 15 MHz was observed. As can be seen in Figure 9c, the average evolution over time of the echo 1 attenuation spectrum for the decontaminated water sample followed that of the reference sample (double distilled water). The elimination of the Sr present in the water using HAp was thus demonstrated. The efficiency of the HAp nanoparticles in the removal of Sr ions was evaluated by ultrasonic analysis based on the temporal evolution of the spectral attenuation of echo 1 relative to bidistilled water. The stability parameter S = A$\frac{dt}{dA}=3.17e-5\left(\frac{1}{s}\right)$ with A(*t*) = signal amplitude, was calculated for the first echo. This definitely demonstrates the effectiveness of HAp in eliminating strontium from the water.

**Figure 10:**
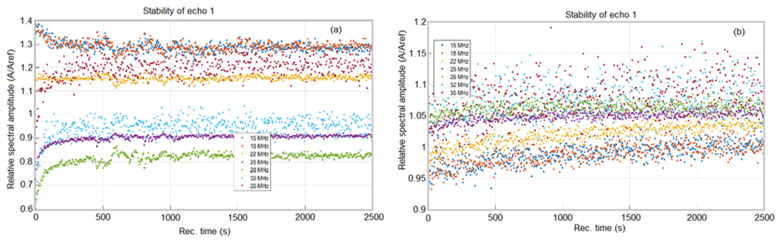
Relative spectral amplitudes versus time for polluted (**a**) and depolluted (**b**) water.

For the remediated water (Figure 10b), the ratio between the 2500 spectral amplitudes calculated for the analyzed suspension and pure water showed very good stability. The weak variations of short duration indicated “clusters” of particles, probably of depolluting powder.

Relative amplitudes around 1 indicate a very low concentration of soluble substances. At very high frequencies, the dispersion of the results is +/−40%, but around the central frequency (25 MHz), the results are clear. In the case of the contaminated water (Figure 10a), low short-term variations were observed indicating “clusters” of particles in suspension which pass through the ultrasound beam. Relative amplitudes >1 indicated the presence of metals (Sr) in the suspension, since a bulk Sr sample would give much larger amplitudes than those passing through water. The results obtained by ultrasound measurement were in good agreement with the XRD studies presented above.

Our studies complement previous studies that demonstrated the efficiency of hydroxyapatite in the removal of radioactive ions, such as Sr^2+^ [57], Co^2+^ [58] and ${\mathrm{UO}}_{2}^{2+}$ [59]. To date, the exact mechanism of removal of divalent metal ions (M^2+^) is not fully understood. Simon et al., in previous studies on “Uranium removal from ground-water using hydroxyapatite”, showed that the mechanism of removal of divalent metal ions (M^2+^) can vary depending on the metal used [59]. Handley-Sidhu et al., in previous studies on “Nano-crystalline hydroxyapatite bio-mineral for the treatment of strontium from aqueous solutions”, suggested that for Sr^2+^, the metal ion could be absorbed on the HAp surface according to the following equations [60]:

OH + M^2+^ → ≡O―M^2+^ + H^+^ (1)

O_3_P-OH^+^ + M^2+^ → ≡O_3_P―O―M^2+^ + H^+^ (2)

Ion exchange:

Ca^2+^ + M^2+^ → ≡M^2+^ +Ca^2+^ (3)

**Figure 11:**
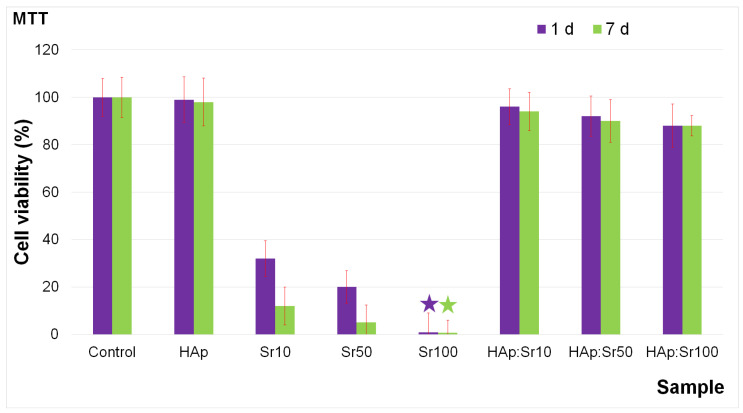
Cell viability of HeLa cells incubated with HAp nanoparticle solution, Sr^2+^-contaminated solutions, and solutions decontaminated using HAp nanoparticles for 1 day and 7 days. HeLa cell culture was used as the control.

The degree to which HAp nanoparticles can cause damage to cells was investigated by in vitro cytotoxicity studies. For this purpose, HAp nanoparticles in solution, and also solutions contaminated with different concentrations of Sr^2+^ ions, as well as decontaminated aqueous solutions using HAp nanoparticles were biologically assessed using HeLa cells. HeLa cells were put into contact for 1 day and 7 days with HAp nanoparticles in solution, as well as with strontium-contaminated solutions; the decontaminated solutions and their viability were evaluated using the MTT assay. The MTT assay results, regarding the HeLa cell viability after treatment with solutions contaminated with strontium ions at different concentrations (10 mg/L (Sr10), 50 mg/L (Sr50), and 100 mg/L (Sr100)) and also with the solutions decontaminated using HAp nanoparticles (HAp:Sr10, HAp:Sr50, and HAp:Sr100), are presented in Figure 11.

The MTT assay demonstrated that the contaminated solutions with various strontium ion concentrations exhibited a strong toxicity towards HeLa cells after 1 day of contact. More than that, the results also emphasized that the HeLa cell viability was significantly determined by the strontium ion concentration. The MTT assay underlined that the cell viability of the HeLa cells was correlated with the strontium ion concentration from the solution. Therefore, a decrease in HeLa cell viability was observed with the increase in the strontium ion concentration. The data suggested that the HeLa cell viability decreased from 32%, in the case of Sr10, to less than 1%, in the case of Sr100-contaminated solutions (green star and purple star in Figure 11). In addition, the outcomes of the MTT assays performed on the aqueous solutions decontaminated using HAp nanoparticles are depicted in Figure 11. The results highlighted that the decontaminated solutions did not present noticeable effects on the cell viability of the HeLa culture after 1 day of contact. Moreover, the MTT assay results demonstrated that for the decontaminated solutions, the HeLa cell viability was above 88%. The data obtained from the MTT assays emphasized that HAp nanoparticles could be considered for the removal of strontium ions from contaminated solutions. Furthermore, the cytotoxicity of the HAp nanoparticles was also evaluated and our results suggest that HAp nanoparticles exhibit no toxicity on HeLa cells after 1 day of incubation. More than that, in order to assess the long term cytotoxic effects of strontium ions against HeLa cells, the cellular viability of the cells was also assessed after 7 days of contact with the HAp nanoparticles, strontium-contaminated solutions and solutions decontaminated using HAp nanoparticles. The results depicted by the MTT studies showed that after 7 days of contact, the HeLa cell viability decreased considerably only in the case of strontium-contaminated solutions. In addition, the results also emphasized that even after 7 days of contact, the decrease in cell viability was correlated with the strontium ion concentration from the aqueous solutions.

**Figure 12:**
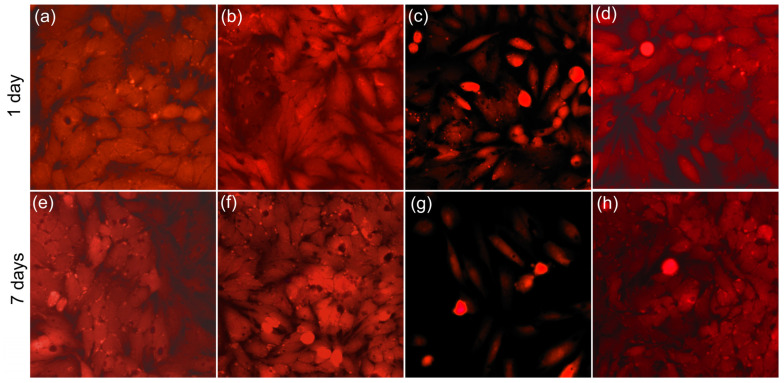
The morphology of the HeLa cells used as control (**a**,**e**); incubated with HAp nanoparticles in solution (**b**,**f**), incubated with Sr^2+^ contaminated solutions (**c**,**g**), and incubated with solutions decontaminated using HAp nanoparticles (**d**,**h**) for 1 day and 7 days.

Supplementary information regarding the cytotoxicity of HAp nanoparticles and the strontium-contaminated and decontaminated solutions was obtained by microscopic observation of the HeLa cells after 1 day and 7 days of contact with the samples. The morphology of the HeLa cells put into contact with the strontium-contaminated solutions at a concentration of 50 mg/L and with the solutions decontaminated using HAp nanoparticles, as well as HAp nanoparticles in solution are depicted in Figure 12.

The optical visualization of the HeLa cells after 1 day of contact with the samples corroborated the results obtained from the quantitative MTT assay and suggested that HAp nanoparticles did not generate any significant morphological changes in the HeLa cells after 1 day of contact. Furthermore, the visualization highlighted that the morphology of HeLa cells was not altered after being exposed to the solutions decontaminated using HAp nanoparticles. More than that, the results of the optical microscopy visualization showed that the morphology of the HeLa cells exposed to the strontium-contaminated aqueous solution was notably modified, indicating that the strontium ions exhibited a cytotoxic effect on the HeLa cells. The HeLa cells exposed to the strontium-contaminated solutions presented distinctive apoptotic features as well as a significant reduction. In addition, the results of the visualization of the HeLa cells put in contact with the samples for 7 days were also in agreement with the MTT assays results and highlighted that both HAp nanoparticles and solutions decontaminated using HAp nanoparticles did not induce any noticeable toxic effects on the HeLa cells. On the other hand, the microscopic visualization depicted that in the case of HeLa cells incubated with strontium-contaminated solutions, a strong decrease in number was observed as well as major morphological changes after 7 days. The cells incubated for 7 days with the strontium-contaminated solutions presented both morphological abnormalities and also appearances characteristic of apoptotic cells. The outcomes, obtained from the microscopic visualization, are in good agreement with the MTT cytotoxicity assay results and with previously reported data regarding strontium toxicity [10,12,61,62,63,64,65,66,67,68,69]. Even though, by now, harmful effects due to overdosing of Sr have not been officially reported in humans, it was demonstrated that the intravenous administration of high doses of Sr induces hypocalcemia due to an increase in, and the renal excretion of, calcium [62]. More than that, in their studies regarding the toxicity assessment of artificially added zinc, selenium, and strontium in water, Liu et al. [12] demonstrated that the 60-day mortality of zebrafish was 100% for a Sr^2+^ concentration of 10 mg/L. In addition, Liu et al. [12] also investigated the mortality of zebrafish caused by different grouping concentrations of Sr^2+^, and their findings determined the values for the maximum non-lethal concentration (MNLC) to be 1.85 mg/mL and the 1/10 lethal concentration of Sr^2+^ to be 1.98 mg/mL. On the other hand, Pasqualetti et al. [69], in their studies regarding the consequences of strontium on the skeletal development in zebrafish embryo, determined that the vitality percentage of the embryos progressively decreased from 86 to 62% for a 5 mM strontium concentration. More than that, for this strontium concentration, embryo modifications were also reported, such as the failure of swim bladder inflation and a lethargic state. The data presented by Pasqualetti et al. [69] also determined that, at a concentration of strontium of 10 mM, the mortality of the embryos was 100% and was achieved in the first 48 h of the experiment. Even though the exact lethal mechanism of toxicity to zebrafish was still unclear, the results of this study highlighted the severe effect of strontium ions in water and made a first step towards understanding the role of strontium ions in the ecosystem. More than that, in their study, Aimaiti et al. [70] determined that a low dose of SrRan helped to enhance hASC osteogenic differentiation, while higher concentrations led to hASC apoptosis. Furthermore, Arkin et al. [71] reported that Sr^2+^ ion-doped cement with a sustainable release of ions in the intervals of 10 to 100 μg/mL under in vitro conditions showed noticeable toxicity and also induced significant cell stress in mouse gingival fibroblast cells, even for small incubation periods. Moreover, their study showed that after 24 of incubation the cell proliferation was reduced to 25% [71]. Therefore, the preliminary observation regarding the cytotoxicity of HAp nanoparticles and strontium ions combined with the good results obtained for the efficiency of HAp nanoparticles in eliminating strontium ions from contaminated solutions validated that these nanoparticles may be appropriate for the development of innovative environmental remediation technologies.

## 4. Conclusions

The present study was focused mainly on the characterization of hydroxyapatite with a high surface area obtained at room temperature and a pH equal to 14 through a simple method developed in laboratory. HAp was studied both before and after its use in the elimination of Sr^2+^ ions from aqueous contaminated solutions. The EDX studies conducted on the HAp powders after the decontamination experiments evidenced the presence of strontium and its homogeneous distribution in the sample. The mean particle size obtained by SEM measurements for the HAp nanoparticles was about 12.5 nm. Furthermore, in the FTIR spectra of the HAp sample recovered after the Sr^2+^ adsorption experiments, the presence of NO_3_^2-^ vibrational bands was noted; a fact that indicates the existence of a secondary phase in the studied sample. On the other hand, the HAp nanoparticle capacity for strontium adsorption from aqueous solutions was studied using batch adsorption experiments. Our studies pointed out an HAp removal efficiency (R%) of 91% for an initial Sr^2+^ concentration above 20 mg/L. The two most common isotherm models, Langmuir and Freundlich, were used to analyze the experimental equilibrium sorption data for the studied sorption process. From the Langmuir adsorption model, a value of 93.63 ± 3.25 mg (Sr)/g for the adsorption capacity was obtained. The adsorption of strontium ions by HAp nanoparticles was very well fitted by the Langmuir adsorption isotherm model, indicating that the HAp nanoparticles presented a high affinity towards strontium ions and had successfully eliminated them from the contaminated solutions. Ultrasound measurements were carried out to verify the stability of the aqueous solution after the removal of Sr^2+^ ions by comparison with bidistilled water taken as the reference solution (known to have the best stability). Furthermore, the cytotoxic effects of HAp nanoparticles and strontium-contaminated and decontaminated solutions were determined using HeLa cells. The biological assays revealed that neither the HAp nanoparticles nor the decontaminated solutions had a toxic effect on the HeLa cells and also that they did not induce any major morphological changes in the HeLa cells after 1 day and 7 days of exposure. Moreover, the quantitative MTT assay highlighted that HeLa cell viability decreased to under 1% when exposed to solutions contaminated with a high strontium concentration. Moreover, for the decontaminated solutions, a cell viability above 88% was obtained after 1 day of incubation. In addition, the optical visualization also emphasized that the strontium-contaminated solution induced major changes in the HeLa cells’ morphology. In conclusion, we can say that the hydroxyapatite that we obtained after the synthesis could be successfully used in the process of removing strontium from aqueous solutions, which would contribute to the management of nuclear waste.

## Figures and Tables

**Table 1:** Langmuir and Freundlich isotherm parameters for Sr^2+^ adsorption onto HAp nanoparticles.

Sample	Langmuir			Freundlich		
HAp	*R* ^2^	*q_m_* (mg/g)	*K_L_* (L/mg)	*R* ^2^	*n*	*k_f_*
	0.997	93.63 ± 3.25	78.19	0.993	4.53	137.37

## Data Availability

Not applicable.
